# The putative role of environmental aluminium in the development of chronic neuropathology in adults and children. How strong is the evidence and what could be the mechanisms involved?

**DOI:** 10.1007/s11011-017-0077-2

**Published:** 2017-07-27

**Authors:** Gerwyn Morris, Basant K. Puri, Richard E. Frye

**Affiliations:** 1 Tir Na Nog, Bryn Road seaside 87, Llanelli, Wales SA15 2LW UK; 2 Department of Medicine, Imperial College London, Hammersmith Hospital, London, England W12 0HS UK; 3 College of Medicine, Department of Pediatrics, University of Arkansas for Medical Sciences, Arkansas Children’s Hospital Research Institute, Little Rock, AR 72202 USA

**Keywords:** Brain, Neuropathology, Aluminum, Alzheimer disease, Autism spectrum disorder, Autoimmunity

## Abstract

The conceptualisation of autistic spectrum disorder and Alzheimer’s disease has undergone something of a paradigm shift in recent years and rather than being viewed as single illnesses with a unitary pathogenesis and pathophysiology they are increasingly considered to be heterogeneous syndromes with a complex multifactorial aetiopathogenesis, involving a highly complex and diverse combination of genetic, epigenetic and environmental factors. One such environmental factor implicated as a potential cause in both syndromes is aluminium, as an element or as part of a salt, received, for example, in oral form or as an adjuvant. Such administration has the potential to induce pathology via several routes such as provoking dysfunction and/or activation of glial cells which play an indispensable role in the regulation of central nervous system homeostasis and neurodevelopment. Other routes include the generation of oxidative stress, depletion of reduced glutathione, direct and indirect reductions in mitochondrial performance and integrity, and increasing the production of proinflammatory cytokines in both the brain and peripherally. The mechanisms whereby environmental aluminium could contribute to the development of the highly specific pattern of neuropathology seen in Alzheimer’s disease are described. Also detailed are several mechanisms whereby significant quantities of aluminium introduced via immunisation could produce chronic neuropathology in genetically susceptible children. Accordingly, it is recommended that the use of aluminium salts in immunisations should be discontinued and that adults should take steps to minimise their exposure to environmental aluminium.

## Introduction

Autism spectrum disorder (ASD) refers to an increasingly common group of heterogeneous disorders identified by the presence of impairments in social interactions and communication together with a restrictive range of repetitive and stereotypical behaviours (Zhubi et al. 2014; Ladd-Acosta et al. 2014). Recent analyses have consistently shown that the prevalence of ASD is increasing. Estimates vary between one in 68 (CDC) and one in 46 (Pelly et al. 2015) to one in 38 (Kim et al. 2011) during similar time periods. There is some debate as to the reasons for the precipitous increase in prevalence of ASD over the past two decades, with some concluding that this is essentially an artefact stemming from the development of broader diagnostic categories and increased medical awareness (Rutter 2005; King and Bearman 2009). However, analyses conducted by other research teams have suggested that other factors aside from diagnostic shift may contribute to this increase in prevalence (Grether et al. 2009; Hertz-Picciotto 2009). Although early heritability estimates for ASD were high (approximately 97%), multiple comprehensive genetic studies have failed to reveal reproducible candidate genes, and thus far no single gene can account for more than 1% of ASD cases, although there is a great deal of evidence that genetic abnormalities play a major role in the development of the disorders which is unsurprising given that this is the case with most if not all illnesses (Siniscalco et al. 2013). It is also worth noting that only a minority of children with an ASD diagnosis have an identifiable genetic syndrome (Siniscalco et al. 2013) and recent studies suggest that the yield of genetic testing is low even when chromosomal microarray analysis is combined with whole exome sequencing, particularly in non-dysmorphic ASD children (Tammimies et al. 2015). Moreover, some genetic abnormalities which increase the risk of developing ASD are also risk factors for other neurodevelopmental or neuroimmune conditions, and are polymorphisms rather than deleterious mutations, being also present in unaffected individuals (Betancur 2011; Sahoo et al. 2011). It would appear that these genes appear to confer vulnerability to a variety of neurodevelopmental disorders (Betancur 2011; Sahoo et al. 2011; Rapoport et al. 2012). Furthermore, a recent heritability estimate stemming from the largest twin study to date yielded a figure as low as 37% (Hallmayer et al. 2011), although another recent study calculated an estimate of heritability between 40 and 60% (Klei et al. 2012).

The concept of ASD as an illness of purely genetic origin has given way to the view that, at the very least, the aetiopathogenesis of ASD involves a highly complex interaction between numerous genes and environmental risk factors (Bushnell 2013; LaSalle 2013). Moreover, it is becoming increasingly apparent that alterations in the epigenetic landscape and dysregulation of epigenetic mechanisms responsible for gene expression also play a major role in the aetiopathogenesis of these disorders (Rangasamy et al. 2013; Flashner et al. 2013; Siniscalco et al. 2013). In a landmark post-mortem microarray study, Voineagu and fellow workers identified 444 genes which were differentially expressed in the cerebral cortex, and two genes which were differentially expressed in the cerebellum, of children with ASD compared with neurotypical age- and sex-matched controls. They reported that the dysregulated patterns of expression of immune and glial gene markers were not associated with any known ASD risk genes, so that immune changes are likely to be either secondary phenomena or the result of environmental factors (Voineagu et al. 2011). In another study, Melnyk and others examined 68 ASD subjects, 40 unaffected siblings and 54 age- and sex-matched controls, and reported oxidative damage to DNA (indexed by the oxidised DNA adduct 8-oxo-deoxyguanosine) and proteins in leukocytes combined with global DNA hypomethylation that was specific to ASD children (Melnyk et al. 2012). The phenomenon of global DNA hypomethylation in the brain as a driver of altered gene expression in ASD children has also been reported (Ladd-Acosta et al. 2014; Nardone et al. 2014). It is also of interest that the latter team reported hypomethylation and consequent upregulation of complement and tumour necrosis factor-alpha (TNF-α) genes, which are involved in the regulation of the neurodevelopmental roles of microglia and synaptic scaling respectively (Nardone et al. 2014).

Further evidence emphasising the importance of epigenetic abnormalities in the pathogenesis of ASD was published by Wong and fellow workers (2014). These authors reported differently methylated DNA regions within 50 monozygotic twin pairs (i.e. 100 individuals) discordant for ASD that correlated with the severity of autistic trait scores, emphasising the importance of epigenetic rather than genetic factors in the pathophysiology and pathogenesis of ASD (Wong et al. 2014). The role of epigenetic dysregulation as an important factor in the pathogenesis of ASD is further supported by evidence of dysregulated microRNA (miRNA) expression in ASD children (Mundalil Vasu et al. 2014; Mellios and Sur 2012). A recent post-mortem study cited evidence of hypomethylated and upregulated miR-142 in the frontal cortex (Brodmann area 10) in children with ASD, which is of interest as this miRNA plays a major role in regulating the neurodevelopmental activities of microglia and maintaining them in a quiescent state (Mor et al. 2015; Vaishnavi et al. 2013; Marrale et al. 2014).

When viewed as a whole, it appears that the aetiology of ASD is multifactorial, involving a highly complex and diverse combination of genetic, epigenetic, environmental and immunological factors (Flashner et al. 2013; Herbert 2010; Roberts et al. 2013). Overall, the conceptualisation of ASD has undergone a paradigm shift, and rather than being viewed as a single illness with a unitary pathogenesis and pathophysiology, it is a clinically heterogeneous syndrome with a complex multifactorial aetiopathogenesis (Zhubi et al. 2014; Voineagu et al. 2011; Betancur 2011; Tordjman et al. 2014; Ruggeri et al. 2014; Georgiades et al. 2013). Ultimately, the pathology in any given child likely has its origins in a dynamic interplay between a broad range of different environmental agents, predisposing genetic factors and complex epigenetic mechanisms as discussed above (Zhubi et al. 2014; Voineagu et al. 2011; Betancur 2011; Tordjman et al. 2014; Ruggeri et al. 2014; Georgiades et al. 2013).

The view of ASD as an illness or illnesses exclusively affecting the brain is also changing. While many ASD children display evidence of activated microglia and astrocytes, which are characteristic of many neuroimmune and neurodegenerative diseases (Morgan et al. 2012; Suzuki et al. 2013; Morgan et al. 2010; Morris and Berk 2015; Morris et al. 2015a), there is also copious evidence of abnormalities in the peripheral immune system. Such evidence includes data demonstrating excessive pro-inflammatory cytokine (PIC) expression, reduced anti-inflammatory cytokine expression, modulated or increased T-cell responses, altered natural killer T-cell responses, activated complement responses, major histocompatibility complex (MHC) class I abnormalities and increased autoantibodies in the periphery as well as in the brain (reviewed in Noriega and Savelkoul 2014; Careaga and Ashwood 2012; Estes and McAllister 2015; Gottfried et al. 2015). The pattern of single nucleotide polymorphisms (SNPs) in immune genes is similar to those seen in several autoimmune diseases such as multiple sclerosis (MS) (Ramos et al. 2012). There is also evidence of abnormally robust pattern recognition receptor activity linked to the presence of SNPs in encoding genes leading to exaggerated immune responses (Enstrom et al. 2010; Mead and Ashwood 2015). It is also noteworthy that genes governing immune and inflammatory responses are upregulated in some children with an ASD diagnosis (Koufaris and Sismani 2015) and that the presence of such abnormally expressed genes can predict the development of ASD in male children with some 83% accuracy (Pramparo et al. 2015). It is also of interest that polymorphisms in cytokine and HLA genes are associated with unusual responses to vaccines (Castiblanco and Anaya 2015). The evidence of immune abnormalities in many, but by no means all, children afforded an ASD diagnosis has led to the proposal of a neuroimmune subtype of ASD (McDougle et al. 2015). Furthermore, several researchers have reported an association between initial inflammatory insults and the subsequent development of chronic immune disturbances in ASD children (McDougle et al. 2015; Gottfried et al. 2015; Siniscalco et al. 2013). One possible explanation for this phenomenon is that an unusually potent and/or prolonged immune response allows for the development of macromolecular or tissue damage leading to the formation of damage-associated molecular patterns (DAMPs) (Lucas et al. 2015). The formation of such DAMPs and the resultant chronic stimulation of pattern recognition receptors (PRRs), leading to the development of an “autotoxic loop” of increasing inflammation and oxidative stress, is considered to play a major role in the maintenance and exacerbation of systemic inflammation, neuro-inflammation and neurodegeneration in a range of autoimmune and neurodegenerative diseases such as systemic lupus erythematosus (SLE), MS and Alzheimer’s disease (AD) (Venegas and Heneka 2017; Land 2015). Frequent or prolonged postnatal infections are an obvious source of inflammatory insults and such events appear to be associated with a significantly increased risk of developing ASD (Abdallah et al. 2012; Hadjkacem et al. 2016). However, other environmental agents putatively associated with an increased risk of developing the ASD phenotype, such as organophosphates, mercury and aluminium, also have the capacity to provoke a prolonged and or exaggerated immune response (Eisenkraft et al. 2013; Kern et al. 2016; Shaw and Tomljenovic 2013). Aluminium salts in adjuvant form functioning as DAMPs activate PRRs and exert profound stimulatory effects on innate immune responses (Powell et al. 2015) and thus could be candidates for the generation of tissue damage and DAMP formation in children with an underlying tendency to produce an exaggerated immune response. There is also evidence of increased aluminium levels in the hair and urine of ASD children compared with unaffected controls (Yasuda and Tsutsui 2013; Mohamed Fel et al. 2015; Blaurock-Busch et al. 2012; Blaurock-Busch et al. 2011), although this not an invariant finding (Fido and Al-Saad 2005; Al-Ayadhi 2005). It should also be noted that aluminium adjuvants are becoming a recognised trigger of autoimmune pathology in genetically susceptible individuals (Morris et al. 2015b).

Intriguingly, chronic or cumulative exposure to aluminium reflected by increased levels in cerebrospinal fluid (CSF) and serum may be one environmental factor in the pathogenesis and pathophysiology of MS, Parkinson’s disease (PD) and AD (Fulgenzi et al. 2014; Exley et al. 2006; Ahmed and Santosh 2010; Yasui et al. 1992; Exley and Vickers 2014; Basun et al. 1991). There is a wealth of research examining the potential association between increased exposure to environmental aluminium and the development of the last of these illnesses. Indeed, a recent meta-analysis involving eight cohort- and case-controlled studies conducted prior to 2015 involving 10,567 participants concluded that increased aluminium exposure increased the risk of developing AD by some 71% (Wang et al. 2016). This seems a noteworthy finding in the light of evidence indicating that AD is also an aetiologically heterogeneous syndrome (Lam et al. 2013; Morris and Berk 2015) as indeed is the case for PD (Klein and Lohmann 2009; Korczyn and Hassin-Baer 2015) and MS (Paz Soldan and Rodriguez 2002). Hence establishing an association between AD and a single environmental factor in a cross-sectional study when a multiplicity of such factors may be involved in different patients is a difficult enterprise. Much of the *in vivo* evidence examining the mechanisms underpinning the pathological effects of aluminium exposure has been obtained in the area of human and animal research into the pathogenesis of AD. Such evidence includes the induction of oxidative stress, mitochondrial dysfunction, microglial activation and functional dysregulation of microglia (Morris and Berk 2016). This may be highly relevant as many children with ASD also manifest oxidative stress (reviewed in Depino 2013; Rossignol and Frye 2014; Frustaci et al. 2012), mitochondrial dysfunction (reviewed by Goh et al. 2014; Chen et al. 2015; Guevara-Campos et al. 2013) and abnormal microglial activity as discussed above.

In this paper we aim to review the available evidence purporting to establish an association between increased aluminium exposure and an increased risk of developing AD and the evidence aimed at illuminating the potential pathophysiological mechanisms by which aluminium could be an element in the development of the illness in at least some people. The objective of this part of the paper is to inform readers with an interest in the pathogenesis and pathophysiology of ASD who might not be aware of concerns regarding aluminium in the pathogenesis of conditions other than ASD. We also aim to highlight accumulating evidence suggesting that aluminium adjuvants can precipitate serious autoimmune or auto-inflammatory pathology in genetically susceptible people which is a growing area of concern. We will then move on to consider evidence suggesting an association between the increased use of aluminium salt adjuvants and an increased incidence of ASD before moving on to touch briefly on the safety or otherwise of vaccines in people with a predisposition to autoimmunity and a range of polymorphisms in immune genes. This would appear to be appropriate in the light of changes in the conceptualisation of ASD as a syndrome with a multiplicity of potential causes and increasing knowledge regarding the effects of genetic variation in the immune system and the response to vaccines. The remainder of the paper will focus on mechanisms by which increased exposure to aluminium could be an environmental trigger of ASD in at least some children with a range of abnormalities in the performance of their immune systems.

## Section 1. Evidence highlighting the neurotoxic properties of aluminium

### Evidence of an association between chronic aluminium exposure and the development of AD

The *p*-block metal aluminium, which is the third most frequently occurring element in the Earth’s crust, occurs naturally in the ore bauxite, various clays and alumino-silicate minerals, and has a preferred oxidation state of +3. Several authors have reported a strong positive correlation between the level of aluminium in drinking water and the incidence of AD throughout the world including the United Kingdom, Canada, Norway and France (Flaten 2001; Kawahara and Kato-Negishi 2011). The most recently reported association was published by Rondeau et al. (2009), who demonstrated that high daily consumption of aluminium in drinking water was associated with a significantly increased risk of developing mild cognitive impairment or AD in a 15-year longitudinal French cohort study involving 1925 recruits (Rondeau et al. 2009).

There is considerable *in vitro* and *in vivo* evidence demonstrating that aluminium ions inhibit the dephosphorylation of tau, potentiate the development of neurofibrillary tangles (NFTs), cause the accumulation of amyloid beta protein and accelerate the formation of amyloid plaques (Kawahara 2005; Exley 2005). Despite such evidence, the enthusiasm for aluminium as a factor in the pathogenesis of AD waned largely based on data suggesting that aluminium levels were no higher in the brains of AD patients than in healthy controls, and the failure to detect aluminium in NFTs and amyloid plaques in post-mortem tissue at higher levels in AD than in age- and sex-matched controls (Yumoto et al. 2009). In particular, no evidence of increased brain aluminium levels in AD was found, using flameless atomic absorption spectrophotometry, in the study of Jacobs et al. (1989). Furthermore, high aluminium levels in the cores of amyloid or neuritic (senile) plaques have not been reported in several studies variously employing scanning proton microprobe analysis (Lovell et al. 1993), energy-dispersive X-ray microprobe analysis (Jacobs et al. 1989), electron microprobe analysis (Chafi et al. 1991) or nuclear microscopy using particle-induced X-ray emission, Rutherford backscattering spectrometry and scanning transmission ion microscopy (Landsberg et al. 1992). In contrast, increased plaque core aluminium has been reported in AD using an energy-dispersive X-ray microanalytical system (Edwardson et al. 1986) and a method based on inductively coupled plasma mass spectrometry combined with flow injection (Beauchemin and Kisilevsky 1998). Similarly, the findings in relation to increased aluminium in NFTs are inconsistent, with a positive finding using laser microprobe mass analysis (Good et al. 1992), negative findings using electron microprobe and ion microprobe analyses (Chafi et al. 1991) and an intermediate finding (that is, slight increase) again using laser microprobe mass analysis (Lovell et al. 1993). A histochemical study of AD hippocampal neurones reported evidence of aluminium in nucleoli and in NFTs (Walton 2006).

Notwithstanding the above findings, some recent research studies using more sensitive techniques have detected aluminium in the brains of AD patients within plaques, NFTs and elsewhere at significantly higher levels than in age- and sex-matched unaffected controls (Yumoto et al. 2009; Bouras et al. 1997). Furthermore, a number of studies reporting the effects of aluminium exposure in animals have demonstrated the development of AD and Alzheimer-like pathology in rodents (Al-Olayan et al. 2015; Abd-Elghaffar et al. 2005; Sumathi et al. 2015; Lu et al. 2014; Exley and Vickers 2014; Exley and Esiri 2006). Animal studies have also revealed that aluminium administered orally or via injection significantly decreased reduced glutathione levels and the activities of catalase, superoxide dismutase, glutathione peroxidase and glutathione reductase, and increased the levels of nitric oxide (NO), PICs and lipid peroxidation (Sumathi et al. 2015; Al-Olayan et al. 2015). Moreover, histological examination has revealed apoptosis of hippocampal and cerebral cortical neurones and the presence of NFTs, amyloid plaque deposition, Schwann cell degeneration and nerve fibre demyelination (Abd-Elghaffar et al. 2005).

Knowledge regarding the possible mechanisms by which aluminium exposure could provoke some of the characteristic features underpinning the pathophysiology of AD has also evolved. In a recent paper, Zhao et al. (2014) reported on the ability of physiologically realistic levels of aluminium to provoke the aggregation of Aβ42 monomers into dimeric, oligomeric, and ultimately fibrillary structures. This team of authors also cited decreased expression of triggering receptor expressed in myeloid/microglial cells-2 (TREM2) in microglia subsequent to the upregulation of miR-34a as the mechanism underpinning impaired microglial-mediated clearance of Aβ42 peptides from the brain caused by prolonged exposure to aluminium at nanomolar concentrations (Zhao et al. 2014).

Human *in vivo* studies have also reported specific aluminium-related abnormalities in the brains of AD patients. The association between prolonged exposure to environmental aluminium and increased levels of phosphorylated tau subspecies in blood lymphocytes has also been reported in a recent study involving 66 retired aluminium workers (Lu et al. 2014). Moreover, it has been demonstrated that ferritin in plasma from AD patients, particularly those with mild AD, contains significantly higher concentrations of aluminium compared with plasma ferritin from age- and sex-matched controls which, given the pivotal role of this protein in the regulation of metal homeostasis, may be a crucial finding; the finding of a higher level in mild AD compared with severe AD may also point to a first phase in which there is an aluminium overload of ferritin, followed by a phase in which ferritin with reduced functional capacity releases aluminium (De Sole et al. 2013). Interestingly, the capacity of aluminium to disrupt the activity of ferritin and transferrin, with the subsequent disruption of iron homeostasis, has been demonstrated in a series of studies implicating aluminium as a potential causative agent in certain types of breast cancer cells as well as in primary invasive breast cancers and ductal carcinoma *in situ* (Darbre et al. 2013; Darbre et al. 2011; Mannello et al. 2013).

It should also be noted that, until recently, an explanation which could explain the selective effects of AD on various regions of the brain was lacking. However, in a study using electrothermal atomic absorption spectroscopy of the aluminium content of the arterial walls of eight arteries which supply the brain, it was found that aluminium concentration is far higher in the posterior cerebral artery (arteria cerebri posterior), which supplies the hippocampus, in late-stage AD patients than in age- and sex-matched controls (Bhattacharjee et al. 2013). This study is particularly intriguing because, when taken as a whole, the data indicate the presence of biochemical mechanisms in the endothelial cells supplying the cerebral vasculature which enable the binding of aluminium to selected areas such as the hippocampus, known to play a major role in the pathogenesis of the illness (Bhattacharjee et al. 2013).

There is little doubt that the weight of evidence implicating aluminium in the causation of AD in at least some patients is increasing. However, at the current time, despite an analysis using Hill’s causality criteria concluding that aluminium played a causative role in the development of AD (Walton 2014), there is currently no universal consensus on the subject, and it seems reasonable to conclude that there is a correlative link between aluminium and AD but that this association does not currently amount to a causative relationship. There is, however, an accumulating body of evidence suggesting that that aluminium in adjuvant form may provoke systematic and symptomatic autoimmune conditions in genetically susceptible individuals and we will now discuss this phenomenon.

### The involvement of aluminium adjuvants in the development of autoimmune syndrome induced by adjuvants (ASIA)

Evidence demonstrating the development of chronic autoimmune or auto-inflammatory conditions following environmental exposure to aluminium salts, and indeed other adjuvants, is increasingly becoming a cause for concern (Zafrir et al. 2012; Cerpa-Cruz et al. 2013; Jensen-Jarolim 2015; Willhite et al. 2014). Much of this evidence has been presented in the context of the “autoimmune (auto-inflammatory) syndrome induced by adjuvants” (ASIA), which encompasses a broad spectrum of immune-mediated illnesses triggered by exposure to medical, cosmetic or environmental adjuvants such as aluminium salts, silicon compounds or indoor mould (Agmon-Levin et al. 2009). ASIA is characterised by specific and non-specific manifestations of autoimmune disease such as chronic fatigue, myalgia, arthralgias, neurocognitive impairment, respiratory symptoms, gastrointestinal symptoms, dermatological signs and the development of autoantibodies (Israeli 2012).

Medical conditions considered by some to be part of the syndrome include post-vaccination phenomena, Gulf War syndrome, macrophagic myofasciitis, antiphospholipid syndrome, siliconosis and possibly chronic fatigue syndrome (myalgic encephalomyelitis) and fibromyalgia syndrome (Cruz-Tapias et al. 2013; Vera-Lastra et al. 2013). It is interesting to note that data from animal models suggest that adjuvants may play a role in the development of syndromic autoimmune diseases such as SLE, Sjögren’s syndrome and rheumatoid arthritis in some patients (Cruz-Tapias et al. 2013; Bagavant et al. 2014).

Adjuvants were once thought to pose little or no independent threat as drivers of pathology. Unfortunately, studies of animal models and humans have demonstrated the ability of some of them to induce autoimmunity and immune-mediated diseases (Agmon-Levin et al. 2009; Elkayam et al. 2011). The mechanisms underpinning adjuvant-induced immunotoxicity appear to be somewhat varied, but clearly impinge on both innate and humoral immune responses (Marrack et al. 2009; Kool et al. 2008a; Eisenbarth et al. 2008). It must be stated however that adjuvant exposure *per se* does not appear to cause problems for the vast majority of people and the development of ASIA seems to depend on genetic predisposition or as yet undetermined environmental co-factors (Perricone et al. 2013; Esposito et al. 2014; Shoenfeld and Agmon-Levin 2011; Soriano et al. 2015).

Several authors have examined patients diagnosed with autoimmune or other immune-mediated illnesses following hepatitis B virus immunisation (Zafrir et al. 2012; Agmon-Levin et al. 2009; Agmon-Levin et al. 2014). The largest such study evaluated the medical records of 93 patients and reported prevalence rates of different manifestations as follows: neurological 67%; general symptoms 60%; musculoskeletal 60%; gastrointestinal 51%; fatigue 42%, ophthalmological 32%; muco-cutaneous 30%; sleep disturbance 19%; psychiatric 16%; and local reactions 11% (Zafrir et al. 2012). Elevated autoantibody titres were also documented in the sera in 80% of the patients. Several vaccine adjuvants have also been implicated in the development of autoimmune diseases which lie outside ASIA, notably ASD which is a subject that we now consider.

### Aluminium adjuvants in the pathogenesis of ASD

From the perspective of aluminium adjuvants as a potential contributory factor in the development of ASD (Shaw and Tomljenovic 2013), a recent analysis applying Hill’s criteria for establishing causality reported children living in countries with the highest prevalence of ASD appear to have the greatest exposure to vaccine based aluminium. Perhaps more importantly, the increase in exposure to aluminium adjuvants displayed a significant positive correlation with the increased prevalence of ASD in the USA recorded over the last 20 years (*r* = 0.92, *p* < 0.0001). A wider analysis revealed the presence of significant positive correlation between the levels of aluminium in adjuvant form administered to preschool children at around three to four months old and the existing ASD prevalence in seven major Western countries (*r* = 0.89 to 0.94, *p* = 0.0018 to 0.0248) (Shaw and Tomljenovic 2013).

Taylor and colleagues, in a meta-analysis of 10 pre-selected predominantly retrospective studies, reported no causal relationship between a range of mercury containing vaccines and a range of neurodevelopmental conditions such as pervasive developmental delay (PDD), attention-deficit hyperactivity disorder (ADHD), autistic disorder and ASD as diagnosed by several different criteria (Taylor et al. 2014).

It is worth noting that the questions asked by the above two groups are different, with the first focusing entirely on autism using current diagnostic criteria while the second used a range of different case definitions of autism and autism-like conditions and also included children with PDD. Indeed, one large study included in the analysis focused entirely on PDD (Smeeth et al. 2004), while another two focused on general neurodevelopmental conditions (Andrews et al. 2004; Verstraeten et al. 2003). It is also fair to say that the conclusions of another study (DeStefano 2007) have been challenged and a re-analysis of the data has revealed a significant association between a first measles, mumps and rubella (MMR) immunisation before the age of 36 months in African-American males and a diagnosis of ASD (Hooker 2014), although it should be noted that this last study has since been retracted. It would appear that the statisticians involved in the meta-analysis by Taylor et al. (2014) viewed PDD, ADHD and ASD as essentially the same condition, or, the interpretation of this and other data is based on the concept of ASD as a discrete disease entity which is being increasingly called into question (Zhubi et al. 2014; Voineagu 2012; Betancur 2011; Tordjman et al. 2014; Ruggeri et al. 2014; Georgiades et al. 2013). However, in spite of these issues those who believe that adjuvants cause “autism” have one question which currently remains unanswered, namely that if aluminium or other adjuvants *per se* provoke chronic central nervous system and peripheral pathology, why is the prevalence of ASD not far higher than it is now? Given the ubiquity of vaccination one would expect that almost every child would be affected. This fact alone means that adjuvants are highly unlikely to be the main cause of ASD. However, research within the ASIA paradigm and beyond does suggest that aluminium adjuvants, and indeed vaccination *per se*, can cause serious long-term pathology in people with a certain genetic vulnerability, especially in the case of latent or subclinical auto-immune diseases, and we now move on briefly to detail such evidence.

## Section 2. Pathological effects of vaccines in people with a predisposition to autoimmunity

Langer-Gould and colleagues reviewed the medical records of 780 patients with newly diagnosed MS, clinically isolated syndrome (optic neuritis, transverse myelitis, and monofocal or multifocal clinically isolated syndrome) or acute disseminated encephalomyelitis (ADEM) and concluded that vaccines may accelerate or precipitate the transition between subclinical and overt symptomatic autoimmune conditions within the first 30 days post-immunisation, particularly in those aged under 50 years (Langer-Gould et al. 2014). Several other authors have reported an association between the quadrivalent human papilloma vaccine and the development of several autoimmune diseases including Raynaud’s disease, Behçet’s syndrome, type 1 diabetes mellitus and Hashimoto’s syndrome (Arnheim-Dahlstrom et al. 2013; Chao et al. 2012). However, once again it would appear that affected patients displayed signs of subclinical autoimmunity prior to vaccination which may have subsequently triggered active disease (Chao et al. 2012; Arnheim-Dahlstrom et al. 2013). Many research teams reviewing adverse event data have reached similar conclusions (Pellegrino et al. 2015; Petrovsky 2015; Guimaraes et al. 2015). Grimaldi-Bensouda and colleagues found a positive association between a personal and family history of autoimmune diseases and the development of several different autoimmune diseases post-vaccination (Grimaldi-Bensouda et al. 2014). Interestingly, and perhaps reassuringly, a prospective longitudinal case-controlled study examining initially unaffected patients with no evidence of overt or covert autoimmune disease failed to demonstrate any association between vaccination and the development of ADEM or other autoimmune conditions (Scheller et al. 2015). However, as previously noted, there is considerable evidence that vaccines, or more likely vaccine adjuvants, may precipitate specific autoimmune sequelae in genetically or epigenetically vulnerable people (Pellegrino et al. 2015; Petrovsky 2015; Guimaraes et al. 2015). We will now discuss possible mechanisms which may underpin this effect.

### Polymorphisms in human leukocyte antigen (HLA) and Toll-like receptor (TLR) and immune response to vaccination

There is a vast body of data demonstrating that immune and inflammatory responses to vaccines such as MMR are heavily influenced by polymorphisms in the HLA region and in genes encoding effector proteins such as cytokines and PRRs which have the capacity to recognise and become activated by conserved pathogen-associated molecular patterns (PAMPs) to produce immune response molecules such as PICs and interferons (Haralambieva et al. 2013; Lucas and Maes 2013). Examples of PRRs include membrane-bound receptors such as TLRs (e.g. TLR-4) and cytosolic receptors such as retinoic acid-inducible gene (RIG)-like receptors (Kumar et al. 2013). Readers interested in examining the evidence purporting to demonstrate an association between HLA polymorphisms and unusual response to the MMR vaccine are referred to an excellent review by Castiblanco and Anaya 2015. Extensive research has also revealed that immune responses to vaccines *per se* in any given individual are determined by polymorphisms and methylation patterns in the HLA region, cytokine and TLR genes coupled with the composition of the microbiome, the presence of co-infections, and a whole host of environmental variables (review Poland et al. 2013). These observations provide the basis for a mechanism whereby adjuvants could provoke an abnormal response in people with certain polymorphisms and/or methylation patterns in the HLA region, cytokine and TLR genes leading to excessively powerful and/or prolonged immune activity resulting in tissue damage and the generation of DAMPs, such as S100b, with the subsequent development of chronic immune and inflammatory pathology (Lucas and Maes 2013; Lucas et al. 2015). This will be the theme developed in the remainder of this paper. First, however, it is appropriate to consider whether aluminium in environmental or adjuvant form does indeed possess the capacity to generate the range of pathology seen in some children with an ASD diagnosis, which we will now consider.

## Section 3. Chronic aluminium exposure and the development of chronic oxidative stress, mitochondrial dysfunction and gliopathology

### Aluminium exposure provoking PIC and chemokine production

Aluminium salt-containing adjuvants induce the production of the interleukins (ILs) IL-1β, IL-8 and IL-18 in TLR-stimulated dendritic cells and macrophages (Kuroda et al. 2011; Li et al. 2008; Sharp et al. 2009). There are now considerable, albeit *in vitro*, data demonstrating that such activation is dependent upon nucleotide-binding oligomerisation domain-like receptor pyrin domain-3 (NLRP3) inflammasome activation (Kool et al. 2008a; Franchi and Nunez 2008). Aluminium salts can activate the NLRP3 inflammasome via a number of different routes. These include destabilisation of phagosomes, acidification of lysosomes and increases in reactive oxygen species (ROS) levels (Kool et al. 2012; Hornung et al. 2008; Sharp et al. 2009). *In vivo*, aluminium hydroxide appears to induce dendritic cell and T-cell activation at least partly via NLRP3 activation (Kool et al. 2008a; Eisenbarth et al. 2008) although alternative routes such as immunoreceptor tyrosine-based activation motif (ITAM) and interferon response factor 3 (irf3) activation appear to be involved (Kuroda et al. 2011; Marichal et al. 2011). Aluminium adjuvants also provoke an immune response via the generation of DAMPs, notably uric acid and host DNA (outside cell nuclei and mitochondria) (Kool et al. 2012; Kool et al. 2008b). There is a body of evidence demonstrating that uric acid and DNA are released *in vivo* following aluminium hydroxide injection (Marichal et al. 2011; Kool et al. 2008b). Uric acid is a DAMP synthesised during purine nucleotide catabolism whose concentration increases during cellular stress, such as at the site of injection (Kool et al. 2008b). The functional relevance of uric acid levels in increasing T-cell priming and the instigation of humoral immune responses has been repeatedly demonstrated (Kool et al. 2008b; Munks et al. 2010). Both uric acid and aluminium hydroxide can independently activate the NLRP3 inflammasome, inducing the secretion of IL-1β (Kool et al. 2008a; Franchi and Nunez 2008). Uric acid crystals administered in the form of an adjuvant can also induce complement responses and a T helper type 2 (T_h_2) cell differentiation pattern (Kool et al. 2011; Shi et al. 2003). Host DNA released into the intracellular space following cellular necrosis also acts as a DAMP. Testimony to the highly immunogenic nature of double-stranded stranded DNA is shown by data demonstrating that it can be used as a substitute for aluminium hydroxide as a vaccine adjuvant (Marichal et al. 2011). Double-stranded cytosolic DNA is sensed by a number of PRRs including TLR-9, leading to the production of PICs via the activation of nuclear factor kappa-light-chain-enhancer of activated B cells (NF-κB) or interferon-beta (IFN-β) via the activation of irf3 (Stetson and Medzhitov 2006; Thompson et al. 2011).

### Aluminium exposure and the generation of oxidative stress

Oxidative damage as evidenced by increased lipid peroxidation and depleted anti-oxidant defences induced by prolonged aluminium exposure appears to be focused in the prefrontal cortex, cerebellum, hippocampus and brainstem (Yuan et al. 2012; Kumar et al. 2011). It is also noteworthy that several authors have reported a linear relationship between increased cellular levels of aluminium and concentrations of protein carbonyls and S100 proteins (Mannello et al. 2013; Darbre et al. 2013; Darbre et al. 2011). This is of particular interest as these molecules may function as DAMPs and cause chronic stimulation of PRRs and hence be a source of chronic immune activation as discussed above. Increased levels of lipid peroxidation in the brain with the production of malondialdehyde (MDA), 4-hydroxy-2-*trans*-nonenal (HNE or 4-hydroxynonenal (4-HNA)) and thiobarbituric acid-reactive substances (TBARS) following oral administration of aluminium chloride is also a common finding in small-animal studies (Newairy et al. 2009; Albendea et al. 2007; Yuan et al. 2012; Lu et al. 2013).

Chronic aluminium exposure also exerts profound detrimental effects on cellular anti-oxidant defences leading to significantly reduced cellular levels of glutathione transferase, glutathione peroxidase, catalase, superoxide dismutase and reduced glutathione (GSH) (Nampoothiri et al. 2015; El-Demerdash 2004; Yousef 2004; Kumar et al. 2011; Newairy et al. 2009). Aluminium ingestion also decreases GSH levels in human blood samples (Khan et al. 2011). Interestingly, aluminium decreases levels of this thiol by inhibiting the activity of NADPH-dependent isocitrate dehydrogenase in mitochondria and malic enzyme and NADPH isocitrate dehydrogenase in the cytosol (Murakami and Yoshino 2004). This is of importance as depleted levels of these enzymes make cells more sensitive to lipid peroxidation and oxidative mitochondrial DNA damage from singlet oxygen species in an environment of chronic oxidative stress (Kim and Park 2003; Lee et al. 2002; Kochevar 2004). In this context the existence of oxidative damage to mitochondrial proteins and DNA following prolonged aluminium exposure was reported by Sharma and colleagues (Sharma et al. 2013). Aluminium ingestion also leads to increased oxidative stress, markers of lipid peroxidation and decreased GSH levels in the epithelial cells lining the small intestine (Orihuela et al. 2005). This depletion of GSH appears to be affected by reduced activity of GSH synthase, GSH reductase and as yet undelineated changes to the plasma membranes resulting in a reduced influx of GSH from the lumen to the mucosa (Orihuela et al. 2005). This aluminium-induced depletion of GSH impairs the activity of calbindin-D9k resulting in decreased transcellular absorption of calcium ions (Orihuela et al. 2005). Given the positive role played by calcium ions in maintaining epithelial barrier integrity (Ma et al. 2000; Schepens et al. 2009), depletion of GSH could well underpin the increases in intestinal inflammation and intestinal barrier permeability caused by prolonged consumption of aluminium (Pineton de Chambrun et al. 2014).

### Aluminium exposure and the development of mitochondrial dysfunction

Oxidative stress and subsequent mitochondrial dysfunction constitute the major vehicle underpinning aluminium-induced neurotoxicity (for review see Kumar and Gill 2014). Exposure to aluminium ions leads to a significant decrease in the activity of cytochrome C oxidase, NADH and succinate dehydrogenase, and a subsequent decrease in state 3 (ADP stimulated) and state 4 mitochondrial respiration, which are likely caused by conformational changes in these enzymes as a direct result of aluminium ion binding (Dua et al. 2010; Mohan et al. 2009; Mustafa Rizvi et al. 2014; Kumar et al. 2008). Aluminium also impairs mitochondrial biogenesis by decreasing levels of peroxisome proliferator activated receptor gamma co-activator 1α (PGC-1α) activity, either directly or indirectly as a result of inducing elevated levels of oxidative stress (Sharma et al. 2013). Aluminium ions also display the capacity to bind to the phosphate groups of ATP and ADP and inhibit the phosphorylation of the latter molecule and the dephosphorylation of the former, and this together with the capacity to inhibit a wide range of kinase and phosphatase enzymes can grossly impair energy homeostasis (Kawahara and Kato-Negishi 2011).

Aluminium can also impair mitochondrial function indirectly via mechanisms such as the induction of endoplasmic reticulum (ER) stress (Mustafa Rizvi et al. 2014; Johnson et al. 2005). Aluminium also induces mitochondrial dysfunction by provoking release of calcium ions from intracellular stores, and it is noteworthy that aluminium-induced oxidative damage and disruption of calcium ion homeostasis is similar in pattern to that seen in AD (Johnson et al. 2005; Walton 2012). The functional and physical relationship between the ER and mitochondria is well documented in the context of apoptosis, but perhaps under-discussed in the context of ER stress which is sub-lethal to the cell (Vannuvel et al. 2013). In the latter environment, the unfolded protein response in general, and protein kinase RNA-like ER kinase activity in particular, leads to a state of chronic mitochondrial underperformance rather than cellular death. This is a complex area and readers wishing to delve deeper into such mechanisms are invited to consult the work of Rainbolt et al. 2014.

Calcium dyshomeostasis is equally detrimental to mitochondrial function and of vital importance in the maintenance of neural function by matching mitochondrial energy production to demand (Rueda et al. 2014; Llorente-Folch et al. 2013). In particular, modest elevations of calcium ions in the cytosol following increases in neural activity act as the “gas pedal” (or “accelerator”) to increase energy production and maintain ATP homeostasis, hence impaired calcium homeostasis can have profound adverse effects on neural function, even in the absence of frank apoptosis (Gellerich et al. 2013) The adverse effects of aluminium on calcium homeostasis is likely one mechanism involved in aluminium-induced neuropathology and we now turn to a consideration of other mechanisms whereby aluminium exposure could result in the type of astrocytic and microglial dysfunction seen in many children with a confirmed diagnosis of ASD.

**Fig. 1:**
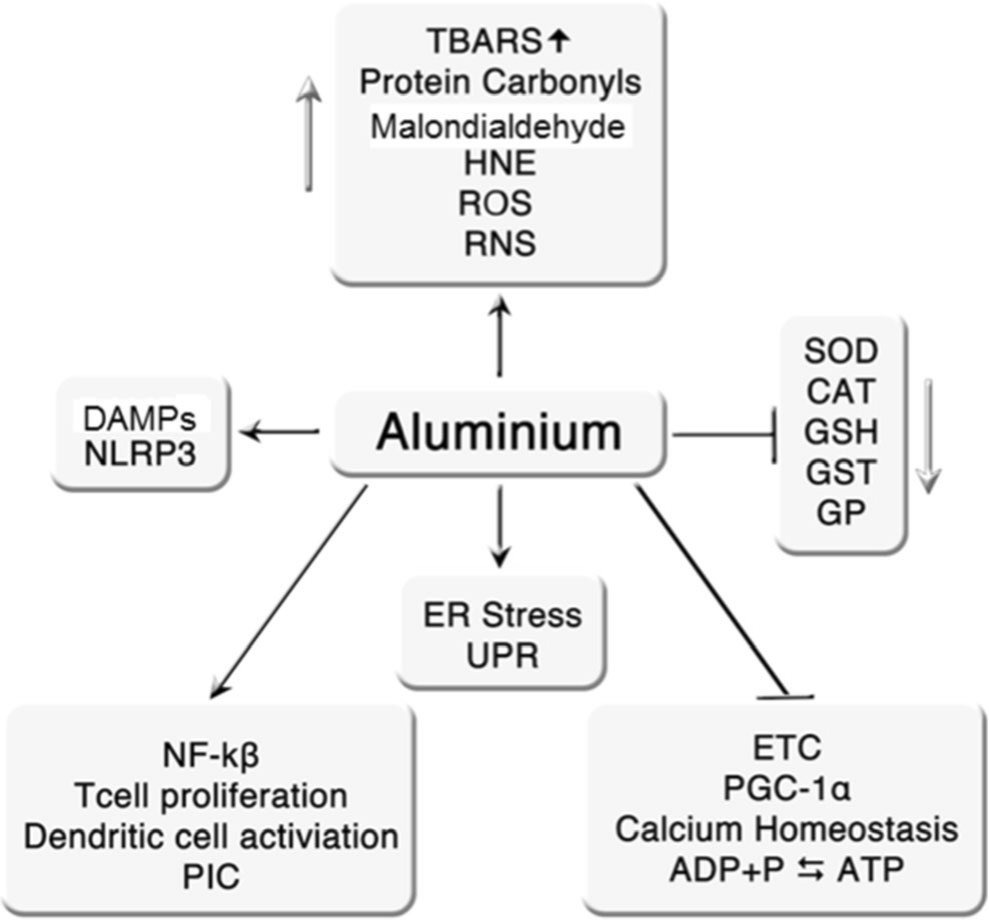
Summary of the effects of aluminium

The above effects of aluminium are summarised in Fig. 1.

### Aluminium exposure and glial cell activation or dysfunction

Chronic or prolonged exposure to aluminium can induce astrocyte apoptosis with one mechanism thought to involve DNA and chromatin damage, and hence mediated by p53 (Suarez-Fernandez et al. 1999; Johnson et al. 2005). Another route may involve inhibition of mitochondrial function and ATP production, ultimately causing necrosis, which can have profound and prolonged neuro-inflammatory consequences (Lemire and Appanna 2011). Prolonged aluminium exposure can also induce significant metabolic changes in astrocytes, which can compromise function even in the absence of degeneration. Such abnormalities include decreasing the activity of γ-butyrobetainealdehyde dehydrogenase and γ-butyrobetainealdehyde dioxygenase and reduced levels of α-ketoglutarate (AKG), leading to low levels of L-carnitine and subsequently impaired fatty acid beta-oxidation, mitochondrial dysfunction, reduced ATP production and increased lipogenesis (Mailloux et al. 2011; Lemire et al. 2011; Han et al. 2013).

The increased lipogenesis subsequent to aluminium-induced mitochondrial dysfunction via this route is enabled by significant increases in lipogenic enzymes such as acetyl CoA carboxylase (Mailloux et al. 2006). The increase in activity of these enzymes is accompanied by decreases in the activity of key enzymes within the electron transport chain and the tricarboxylic acid (Kreb’s) cycle, such as succinate dehydrogenase and AKG, leading to a significant decrease in the levels of ATP produced by oxidative phosphorylation (Mailloux et al. 2009; Mailloux et al. 2006; Mailloux et al. 2007). The loss of AKG activity in mitochondria and cytosol, likely caused by sequestration of this molecule by antioxidant defences, also results in significant negative consequences for energy generation within the glial cells. Briefly, AKG acts to stabilise hypoxia-inducible factor-1-alpha (HIF-1α) in the cytoplasm and prevents its translocation to the nucleus. However, in a cellular environment of increased aluminium cations and subsequently reduced AKG levels, HIF-1α translocates to the nucleus provoking increases in the transcription of hexokinase, pyruvate kinase, lactate dehydrogenase and glucose-6-phosphate dehydrogenase, with the ultimate effect of switching from energy production by oxidative phosphorylation to the phylogenetically more ancient pathway of energy production via glycolysis (Mailloux and Appanna 2007; Agrawal et al. 2007).

Aluminium also induces significant alterations to glutamate/glutamine recycling within astrocytes leading to increased glutamine to glutamate conversion coupled with increased uptake of glutamate and increased intracellular levels of glutamine (Zielke et al. 1993; Struys-Ponsar et al. 2000). This has the effect of modulating glutamatergic and GABA-ergic neurotransmission, but may also have significant bioenergetic consequences given that increased levels of glutamate within astrocytes act as a further stimulus for increased glycolysis (Albrecht et al. 2010; Bouzier-Sore and Pellerin 2013). These observations are pertinent from the perspective of potentially impaired neurodevelopment as astrocytes play an important role in the development of the brain by regulating processes involved in synaptic transmission, neuronal migration, synaptogenesis and maybe even myelination (reviewed by Molofsky et al. 2012). The weight of evidence also indicates that the activity of these glial cells is of paramount importance in the development and maintenance of neural networks and circuits (Clarke and Barres 2013). Furthermore, there is now considerable evidence indicating that impaired astrocyte function plays a pivotal role in the pathogenesis of neurodevelopmental disorders (Molofsky et al. 2012; Sloan and Barres 2013; Yang et al. 2013).

Aluminium can activate microglia leading to secretion of TNF-α, IL-6 and cytokine-inducible nitric oxide synthase (iNOS or NOS-2) and the development of neuro-inflammatory PICs and ROS (Bondy 2010; Zaky et al. 2013). This is also an important finding as there is now overwhelming evidence demonstrating that microglia play an indispensable role in the development of the brain by regulating processes such as synaptic pruning, synaptic plasticity, synaptogenesis, neuronal development and other vital processes in neurogenesis (Kettenmann et al. 2013). Microglial dysfunction and/or priming provoked by immune challenges, inflammatory events or other changes in the brain which interfere with processes such as synaptic pruning and neural proliferation is now thought to play a major causative role in the development of ASD and other neurodevelopmental disorders such a schizophrenia (Harry and Kraft 2012; Harry 2013; Hoeijmakers et al. 2016; Edmonson et al. 2016). The stepwise development of microglia is regulated by the activity of several genes, and disruption in the expression of these genes can occur as a result of prenatal immune activation or disturbances in the microbiota (Matcovitch-Natan et al. 2016). Aluminium can also provoke microglial activation and dysfunction via several other mechanisms, notably by impairing the function of the multifunctional molecular complex of the adapter protein, DNAX activating protein of 12 kD (DAP12) with TREM2, i.e. DAP12/TREM2, which is expressed on the surface of glial cells (Bhattacharjee et al. 2014; Zhao et al. 2014; Alexandrov et al. 2013).

The DAP12/TREM2 protein complex plays a major role in the regulation of central nervous system homeostasis (Paradowska-Gorycka and Jurkowska 2013; Thrash et al. 2009). TREM2 binds a range of liposaccharides, phospholipids and polyanions with the subsequent activation of DAP12 and an array of downstream kinases cumulating in cellular activation (Poliani et al. 2015; Takahashi et al. 2007). Activation of the DAP12/TREM2 axis has an important role in limiting PICs and other neurotoxins following TLR activation by PAMPs and DAMPs, and promotes the survival and proliferation of microglia and other cells of the myeloid lineage (Poliani et al. 2015; Peng et al. 2013). However, in recent years the bulk of research on the DAP12/TREM2 complex has focused on its role in enabling and regulating microglial phagocytosis (Neumann and Takahashi 2007; Painter et al. 2015). TREM2 acts as a molecular sensor of macromolecular debris and plays an indispensable role in its immunogenically silent clearance, thereby resolving potentially inflammatory reactions and acting as an impediment to the development of neuro-inflammation (Painter et al. 2015; Takahashi et al. 2005; Takahashi et al. 2007; Hsieh et al. 2009).

Microglial phagocytic processes play an essential role in removing damaged or stressed neurons and synaptic structures, apoptotic cells and other cellular debris from the brain, which have the potential of being immunogenic, and thus militate against the development of neuro-inflammation. Hence, this process plays an indispensable role in the maintenance of neural homeostasis (Fu et al. 2014; Jones et al. 2014; Zhao and Lukiw 2013). Unsurprisingly, several research teams have reported that failure of these unceasing microglial phagocytic processes have serious and potentially catastrophic innate-immune, pro-inflammatory and neuropathogenic consequences (Jones et al. 2014; Koenigsknecht-Talboo et al. 2008). Crucially, as previously noted, microglial apoptosis is both regulated and enabled by TREM2 (Zhao and Lukiw 2013; Alexandrov et al. 2013; Jones et al. 2014; Wang et al. 2015). Impairment in the activity and/or expression of TREM2 causes gross impairments of microglial phagocytic activity leading to a broad spectrum of central nervous system pathology including neuro-inflammation, synapatic loss, neuronal loss and exaggerated production of PICs (Wang et al. 2015; Hickman and El Khoury 2014; Jiang et al. 2014).

TREM2 expression in turn is regulated by a number of miRNAs, most notably miR-34a (Zhao et al. 2013; Zhao and Lukiw 2013; Alexandrov et al. 2013). Microglial miR-34a expression is upregulated by activation of the pro-inflammatory transcription factor NF-κB (Zhang et al. 2011; Hickman and El Khoury 2014; Bhattacharjee et al. 2014). Aluminium in adjuvant or environmental form upregulates NF-κB (Pogue et al. 2009; Bondy 2014), and thereby induces a number of NF-κB-sensitive pro-inflammatory miRNAs, notably miR-34a (Fu et al. 2014; Jones et al. 2014; Hickman and El Khoury 2014; Zhang et al. 2013; Zhao and Lukiw 2013), which in turn downregulates TREM2 expression in the membranes of glial cells leading to a profound deficit in their phagocytic capability (Hickman and El Khoury 2014; Zhao et al. 2013; Bhattacharjee et al. 2014).

Upregulation of NF-κB induces the synthesis of other inflammatory miRNAs such as miR-9, miR-125b, miR-146a and miR-155, which are recognised drivers of pathology in several neurodegenerative diseases such as AD (Zhao et al. 2014; Lukiw 2012). While aluminium clearly could provoke chronic pathology in people with a high or prolonged exposure, it should be further emphasised that the likelihood that adjuvant use could be the main cause of ASD in children, or indeed harmful to the vast majority of children with normal immune responses, appears to be vanishingly small. However, the situation in children with an abnormal immune system and a predisposition to autoimmunity may be different, and this will be the final area of discussion in this paper.

**Fig. 2:**
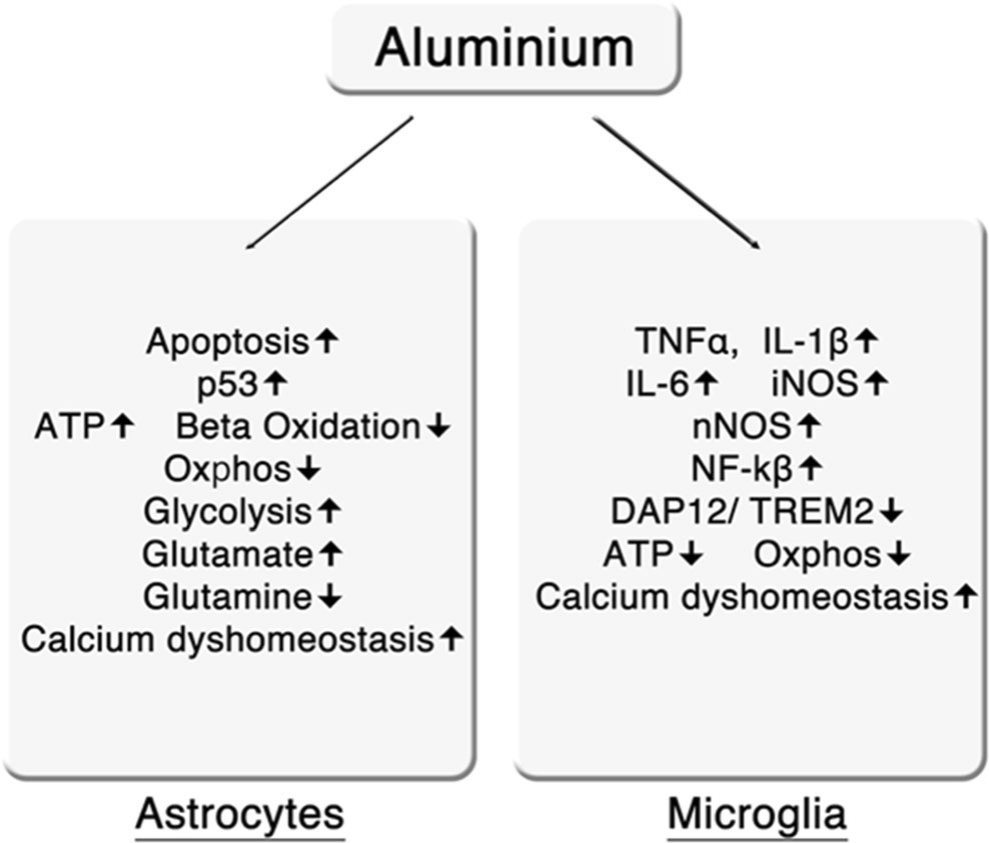
Effects of aluminium on astrocytes and microglia

The actions of aluminium on astrocytes and microglia are summarised in Fig. 2.

## Section 4. Consequences of aluminium exposure in individuals with abnormal immune responses

### Polymorphisms in immune effector genes

Abnormalities in the performance of PPRs are increasingly associated with the development of neurodegenerative and autoimmune conditions (Moraes et al. 2013; Cook et al. 2004; Marshak-Rothstein 2006; Pradhan et al. 2012; Liu et al. 2014). In particular, functional polymorphisms in the genes encoding proteins involved in effecting the immune response following ligation of TLR-4, TLR-7 and TLR-9 increase the susceptibility to and/or severity of a range of neurological, auto-immune, inflammatory and infective illnesses, including SLE, rheumatoid arthritis, asthma, sepsis and hepatitis (Moraes et al. 2013; Dhaouadi et al. 2013; Fichna et al. 2016; Netea et al. 2012). Unsurprisingly, functional polymorphisms in cytokine genes such as those encoding for TNF-α, IL-β, IL-6, IL-10 and IL-4 also increase the susceptibility to develop a range of inflammatory, infective and autoimmune conditions (Morris and Berk 2015).

The presence of these polymorphisms can also heavily influence the severity and ultimate trajectory of these diseases in any given individual (Arfanakis et al. 2013; Tunçbilek 2014; Haukim et al. 2002; Hollegaard and Bidwell 2006). There is also accumulating evidence that functional polymorphisms in genes encoding cytokines and/or their receptors increase the risk of neuro-inflammatory and neurodegenerative pathology such as stroke, AD, and MS (Ottoboni et al. 2013; Bagnoli et al. 2007; Miranda-Hernandez and Baxter 2013; Cui et al. 2012). Functional polymorphisms can also lead to altered structure and function of receptor and effector proteins involved in a broad range of inflammasome responses, and once again such mutations are also involved in the pathogenesis of several autoimmune, neuro-immune and neurodegenerative diseases (Pontillo et al. 2012; Tan et al. 2013; review by Yang and Chiang 2015). In general, “pathological” polymorphisms within nuclear localisation leucine-rich-repeat protein-1 (NLRP1) pathways lead to an exaggerated and/or prolonged inflammatory response (Levandowski et al. 2013).

It is of interest that recent research has revealed a significant association between the cumulative presence of immune gene polymorphisms and increased risk of developing ASD (Ramos et al. 2012). Single nucleotide polymorphisms (SNPs) in immune genes in many children also display a signature pattern which is characteristic of autoimmune diseases such as MS (Jung et al. 2011). Unsurprisingly, polymorphisms in the HLA system, notably HLA-DR4 and HLA-A2, are associated with a significantly increased likelihood of developing the syndrome (Mostafa et al. 2013; Torres et al. 2012). It is also noteworthy that the concomitant presence of abnormally expressed genes governing the regulation and delivery of immune and inflammatory responses can predict the development of ASD with some 83% accuracy (Pramparo et al. 2015).

There is now copious evidence of dysregulated expression of genes regulating the innate and adaptive immune response in children with ASD (Michel et al. 2012; Gupta et al. 2014). There is also some evidence of functional polymorphisms coupled with exaggerated PRR responses in at least some affected children (Bennabi et al. 2015; Enstrom et al. 2010). There is also an accumulating body of research demonstrating that immune responses following activation of TLRs are abnormal in many children who have received a diagnosis of ASD (Mead and Ashwood 2015; Gesundheit et al. 2013; Enstrom et al. 2010). Moreover, several research teams have adduced evidence indicating that the immune response is abnormal in many children with ASD, and in particular that genes governing the regulation and performance of immune and inflammatory responses are upregulated allowing for an excessive and/or prolonged response to an environmental insult (Voineagu and Eapen 2013; Koufaris and Sismani 2015). It is also noteworthy that several researchers have also reported an association between initial inflammatory insults and the subsequent development of chronic immune disturbances in ASD children (Siniscalco 2015; Gottfried et al. 2015; McDougle et al. 2015). This is particularly noteworthy, as prolonged and/or excessive activation of immune and inflammatory pathways leading to oxidative and nitrosative stress can have detrimental consequences for cellular and tissue integrity, which in turn could lead to the chronic activation of immune and inflammatory pathways and ultimately of microglia and astrocytes in the brain as we will now discuss.

### Prolonged or excessive immune responses and the production of DAMPs

Oxidative stress activates NFκB and other transcription factors such as activated protein-1 (AP-1), leading to the synthesis and secretion of PICs, various chemokine species by antigen presentation cells, and activation and proliferation of T and B lymphocytes (Morris et al. 2015a; Lucas et al. 2015). Such activation and PIC production leads to the upregulation of iNOS and NADPH oxidase, leading to the production of superoxide, NO and peroxynitrite, and hence further increases in ROS and reactive nitrogen species (RNS) levels (Morris and Maes 2014; Morris et al. 2015a). This bidirectional and self-amplifying relationship between the development of chronic systemic inflammation as evidenced by increased PIC levels and chronic nitro-oxidative stress as evidenced by increased levels of ROS and RNS is often labelled an “autotoxic loop” (Reuter et al. 2010; Ortiz et al. 2013). Excessive levels of ROS and RNS can also lead to the oxidative and nitrosative modification of lipids, proteins and DNA, leading to misfolded, and consequently immunogenic, proteins, oxidative modification of DNA and lipid peroxidation of cell membranes, together with the production of highly reactive ketones and aldehydes (Lucas et al. 2015). The net result of these processes is the indirect and direct formation of DAMPs capable of activating pathogen-sensing receptors on the cell surface, and in the cytoplasm of immune cells (Hwang 2013; Ortiz et al. 2013).

In summary, the presence of the immune abnormalities discussed allows for an abnormally excessive or prolonged inflammatory response following exposure to aluminium adjuvants, leading to the production of DAMPs and subsequent chronic immune activation in genetically susceptible children. It is noteworthy that several authors have reported the presence of DAMPs such as protein carbonyls, MDA and high-mobility group box-1 (HMGB1) protein in at least some children afforded a diagnosis of ASD, and such DAMPs could play a major role in initiating and maintaining a state of immune activation and inflammation seen in many children with an ASD diagnosis (Babinska et al. 2014; Emanuele et al. 2010; Frank et al. 2016; Napoli et al. 2013).

Chronic engagement of TLRs by DAMPs leads to the development of a positive feedback loop, whereby increasing tissue damage caused by elevated PICs, ROS and RNS perpetuates and escalates pro-inflammatory responses, leading to a state of chronic inflammation nitro-oxidative stress, mitochondrial dysfunction and glial cell activation (Drexler and Foxwell 2010; Piccinini and Midwood 2010; Goh and Midwood 2012; Morris and Berk 2015). Unsurprisingly, chronic engagement of TLRs, nucleotide-binding oligomerisation domain (NOD)-like receptors and RIG-like receptors is implicated in the pathogenesis and pathophysiology of SLE, rheumatoid arthritis and MS (review (Drexler and Foxwell 2010; Piccinini and Midwood 2010; Goh and Midwood 2012)). Pertinently, the presence of DAMPs can also lead to the chronic activation of the inflammasome (Anders and Schaefer 2014) which is also implicated in the development of neuro-inflammation and abnormal central nervous system signalling characteristic of neurodegenerative and neurodevelopmental disorders (Tan et al. 2013; Singhal et al. 2014). It is also of interest that a recent study has reported the presence of activated inflammasome complexes in at least some children afforded a diagnosis of ASD (Saresella et al. 2016) There is now overwhelming evidence that prolonged and/or chronic activation of peripheral immune and inflammatory pathways provokes the development of chronic neuro-inflammtion and/or microglial priming and a brief explanation of this process will constitute the final subsection of this paper.

### Systemic immune activation primed microglia and chronic neuro-inflammation

There is ample evidence demonstrating that chronic immune system activation and systemic inflammation can lead to the development of chronic neuro-inflammation (Perry and Holmes 2014; Cunningham 2013). Communication of inflammatory signals to the brain is mediated by PICs via a number of routes, including innervation of the vagus nerve, carrier-enabled transport across the blood-brain barrier (BBB), activation of endothelial cells within the BBB and perivascular macrophages, and finally via transport through circumventricular organs devoid of a functional BBB (Morris and Maes 2012, 2014). The transduced inflammatory signals may lead to the development of chronic neuro-inflammation via the activation of microglia if of sufficient intensity and/or duration or lead to the development of “primed” microglia (Perry and Holmes 2014; Su and Federoff 2014). Microglial priming involves the upregulation of a range of surface receptors such as MHC class II, CD11b and CD11c integrins, co-stimulatory molecule CD86 and TLR-4 (Su and Federoff 2014).

Following the upregulation of these receptors, such microglia become exquisitely sensitive to subsequent inflammatory stimuli, leading to an exaggerated production of neurotoxic molecules that may exacerbate the pre-existing pathology and may even accelerate the progression of existing neuro-inflammatory or neurodegenerative diseases (Ferrari and Tarelli 2011; Lunnon et al. 2011). Activated microglia exert their neurotoxic effects by releasing PICs, such as TNF-α, IL-1β, IL-6, and IFN-γ, and free radicals including superoxide, NO and peroxynitrite, as well as inflammatory molecules such as prostaglandin E2. Moreover, TNF-α, IL-1β and IFN-γ can act as secondary sources of RNS and other inflammatory molecules by acting as potent inducers of iNOS and via their capacity to upregulate cyclooxygenase-2 (COX-2) with the resultant production of prostaglandin E2 (Morris et al. 2015b; Perry et al. 2010). The concept of microglial priming could change the frame of reference from a consideration of a single inoculation containing aluminium adjuvant to a cumulative effect caused by a vaccination schedule in which successive immune insults over a short period could provoke chronic pathology either directly, by provoking microglial activity, or more indirectly by provoking macromolecular damage which could eventually reach a threshold capable of provoking chronic pathology. It should be noted that there is an accumulating body of evidence, albeit from animal studies, that successive and frequent postnatal immune and inflammatory insults play a pivotal role in the advent of microglial priming and the genesis of neurodevelopmental disorders (Ibi and Yamada 2015; Nagai 2013). There is also emerging data implicating the development of microglial priming as a major factor in the development of several if not all neurodegenerative diseases (Bhattacharya et al. 2016; Zhao et al. 2014; Shastri et al. 2013; Hoeijmakers et al. 2016).

## Summary

Evidence of the neurotoxicity of aluminium cations (Al^3+^) includes: an association between chronic aluminium exposure and the development of AD; the involvement of aluminium adjuvants in the development of ASIA; and epidemiological evidence pointing to an association between the use of aluminium adjuvants and ASD. There is good evidence to suggest that immunisation may accelerate or precipitate the transition between subclinical and overt symptomatic autoimmune conditions within the first 30 days post-immunisation, particularly in those younger than 50 years of age. The immune response to immunisation may be influenced by variations in HLA, TLR and cytokine genes. Moreover, aluminium exposure is associated with the production of pro-inflammatory cytokines and chemokines and with the development of chronic oxidative stress, mitochondrial dysfunction and glial activation or dysfunction; these changes in turn are associated with ASD.

## Conclusions and Future Directions

Aluminium has no known beneficial physiological action in the human body and some genetic polymorphisms predispose to a greater susceptibility to its adverse effects. Therefore, a strong case can be made for avoiding unnecessary exposure to environmental sources of aluminium salts, especially on the part of children, pregnant mothers and women of child-bearing age who may become pregnant. Such avoidance need not lead to hardship or inconvenience; aluminium cookware may be replaced by safer alternatives, while aluminium-containing antiperspirants, potentially implicated in the rise of cases of breast cancer particularly affecting the upper outer quadrant of the mammary gland, may be replaced by non-aluminium versions. The use of aluminium salts in medical products is a more contentious issue. While antacids are available which do not contain aluminium salts, the avoidance of immunisations which do not contain aluminium salts as adjuvants has wider political and financial implications. It would seem prudent to try to find an alternative to aluminium adjuvants as soon as possible and phase out their use.
